# Adenocarcinoma with fetal features invading the right superior sulcus treated with neoadjuvant chemoradiotherapy followed by complete video-assisted thoracoscopic right upper lobectomy: a case report

**DOI:** 10.1186/s40792-019-0737-z

**Published:** 2019-10-29

**Authors:** Kensuke Kojima, Hyungeun Yoon, Tetsuki Sakamoto, Tomoki Utsumi, Teiko Sakurai, Naoko Takeuchi, Maiko Takeda, Takahiko Kasai, Shinji Atagi, Akihide Matsumura

**Affiliations:** 1 Department of General Thoracic Surgery, National Hospital Organization Kinki-Chuo Chest Medical Center, 1180 Nagasone-cho, Kita-ku, Sakai-shi, Osaka 591-8555 Japan; 2 Department of Internal Medicine, National Hospital Organization Kinki-Chuo Chest Medical Center, 1180 Nagasone-cho, Kita-ku, Sakai-shi, Osaka 591-8555 Japan; 3 Department of Pathology, National Hospital Organization Kinki-Chuo Chest Medical Center, 1180 Nagasone-cho, Kita-ku, Sakai-shi, Osaka 591-8555 Japan

**Keywords:** Adenocarcinoma with fetal features, High-grade fetal adenocarcinoma of the lung, Superior sulcus tumor, Complete video-assisted thoracoscopic surgery

## Abstract

**Background:**

Fetal adenocarcinoma of the lung is a rare lung neoplasm that accounts for only 0.5% of all primary lung cancers. Because of its rarity, effective treatments for the management of the tumor are poorly understood. We herein report a case of adenocarcinoma with fetal features of the lung with invasion of the right superior sulcus that was treated with neoadjuvant chemoradiotherapy followed by surgical resection.

**Case presentation:**

A 54-year-old man was referred to a medical institution due to right inner forearm pain. On computed tomography of the chest, a 56-mm mass with invasion of right superior sulcus was discovered. Bronchoscopic biopsy revealed non-small cell lung carcinoma. We performed concurrent chemotherapy (2 cycles of cisplatin and vinorelbine) and thoracic radiation therapy (40 Gy in 20 fractions). As the result of extreme tumor reduction after neoadjuvant chemoradiotherapy, we could perform right upper lobectomy by complete video-assisted thoracoscopic surgery. Since no viable cancer cells were detected from the pathological examination of the resected tissue, the specimen obtained by bronchoscopic biopsy was reexamined by immunohistochemistry. The analysis supported a pathologic diagnosis of adenocarcinoma with fetal features.

**Conclusions:**

We experienced a case of adenocarcinoma with fetal features of the lung in which the patient showed a complete response to neoadjuvant chemoradiotherapy. In addition, the tumor invading the right superior sulcus was completely resected by video-assisted thoracoscopic lobectomy. Neoadjuvant chemoradiotherapy followed by surgery may be also an effective treatment for advanced-stage high-grade fetal adenocarcinoma of the lung, similarly to other subtypes of advanced-stage primary lung cancer.

## Background

Fetal adenocarcinoma of the lung (FLAC), which is classified into low-grade (L-FLAC) and high-grade (H-FLAC), is an extremely rare subtype of pulmonary tumor occurring with a relative incidence of 0.5% among all lung cancers [1]. Because of its rarity, optimal treatments for the management of the disease are poorly understood. We report a case of H-FLAC with invasion of the right superior sulcus that showed a pathologic complete response to neoadjuvant chemoradiotherapy followed by right upper lobectomy performed by complete video-assisted thoracoscopic surgery (VATS).

## Case presentation

**Fig. 1:**
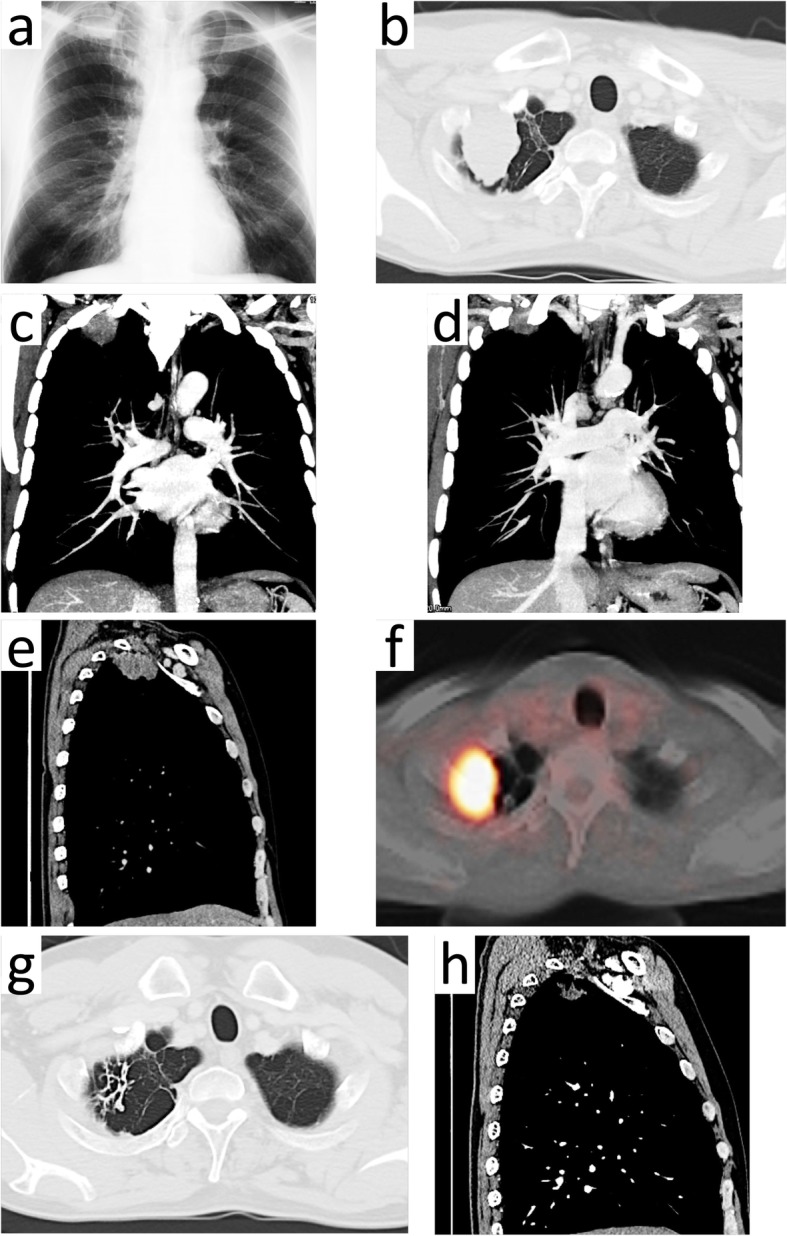
Radiologic imaging of the chest. **a** Chest X-ray showed a tumoral shadow at the right apex of the upper-lung field. **b** Computed tomography (CT) of the chest (pulmonary window): a 56 × 30 × 25-mm mass was identified at the right superior sulcus. **c**, **d** Coronal chest CT (mediastinal window): the mass was suspected to have invaded the chest wall at the right first and second intercostal space and the subclavian vein. **e** Sagittal chest CT before chemoradiotherapy (mediastinal window). **f** Fluorodeoxyglucose-positron emission tomography (FDG-PET). The mass showed a high FDG uptake (SUVmax 13.5). **g** Chest CT (pulmonary window) performed after chemoradiotherapy revealed the remarkable reduction of the tumor. **h** Sagittal chest CT after chemoradiotherapy (mediastinal window). The reduction of the mass to 19.5 mm in long diameter was recognized. In addition, it seemed that the contact between the tumor and the chest wall had been released

A 54-year-old Japanese man with a 30 pack-year smoking history was referred to a medical institution due to pain and paresthesia from the ulnar surface of the right forearm to the small finger of the right hand in the distribution of the C8 and T1 dermatomes that had persisted for approximately 2 months. He had no remarkable medical history. The patient’s laboratory data revealed an elevation carcinoembryonic antigen (CEA) level of 8.4 ng/ml. A chest X-ray showed a tumoral shadow at the right apex of the upper-lung field. (Fig. 1a). On a computed tomography (CT) of the chest, a 56 × 30 × 25-mm mass was discovered at the right superior sulcus (Fig. 1b–e). The mass was suspected to have invaded the chest wall of the right first and second intercostal space (Fig. 1b–d). Additionally, the disruption of blood flow of the subclavian vein, which was suspected to be due to tumor invasion, was confirmed (Fig. 1d). Fluorodeoxyglucose-positron emission tomography (FDG-PET) demonstrated high FDG uptake (SUVmax 13.5) in the mass at the right lung apex (Fig. 1f). Bone scintigraphy and cerebral magnetic resonance imaging (MRI) showed no evidence of distant metastasis. The histopathological diagnosis of a bronchoscopic biopsy specimen was non-small cell lung cancer-suspected adenocarcinoma. The tumor was classified as cStage IIB (cT3N0M0). The patient was referred to our hospital for the treatment. We decided to perform neoadjuvant chemoradiotherapy followed by right upper lobectomy.

We induced chemotherapy with cisplatin 80 mg/m^2^ on day 1, plus vinorelbine 20 mg/m^2^ on days 1 and 8. Two cycles of chemotherapy were scheduled every 21 days. Concurrent thoracic radiation therapy (40 Gy in 20 fractions of 2 Gy each) was performed. Chest CT after chemoradiotherapy indicated remarkable reduction of the tumor (Fig. 1g, h). Also, it revealed a partial response in which tumor volume decreased by 65.2% (Fig. 1h). In addition, most of the contact between the tumor and the chest wall was released (Fig. 1h). We assessed the patient at stage ycT1bN0M0 and ycStage IA2. The pain and paresthesia from the ulnar aspect of the right forearm to the little finger were much improved. The laboratory data showed that the CEA level had decreased to 6.9 ng/ml. Because there was no evidence of distant metastasis or local progression, we planned to perform right upper lobectomy 4 weeks after the completion of the chemoradiotherapy. Because chest CT after chemoradiotherapy indicated the remarkable shrinkage of the tumor, we decided to attempt thoracoscopic right upper lobectomy. When firm adhesion is observed between the tumor and chest wall, our policy is to perform open thoracotomy with chest wall resection.

**Fig. 2:**
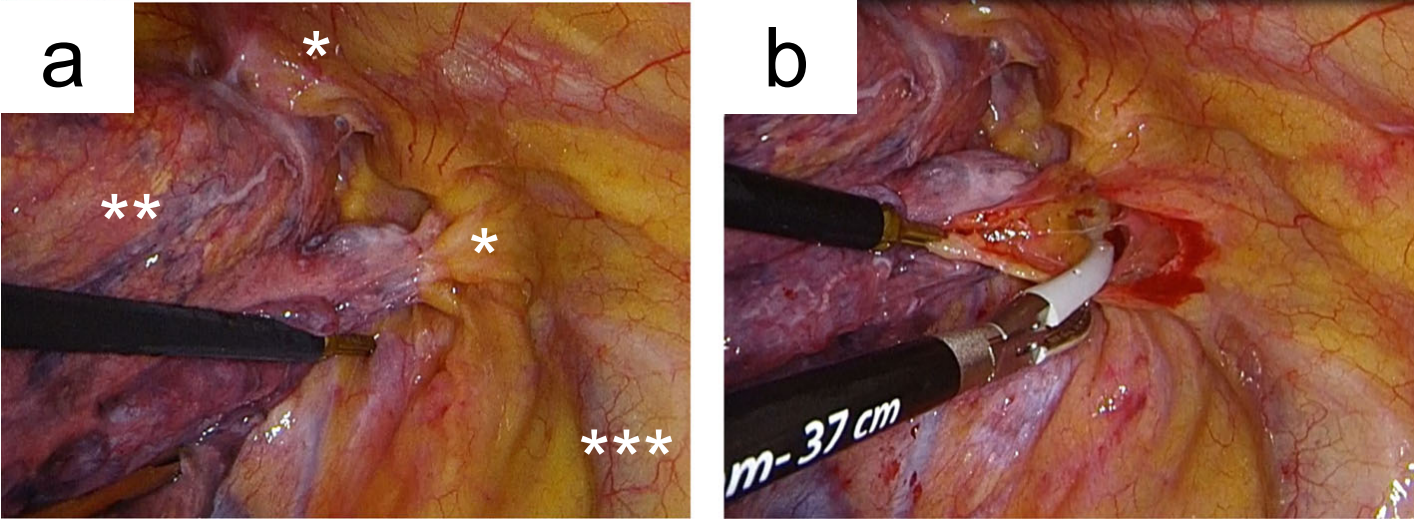
Thoracoscopic views of the right superior sulcus. **a** Partial parietal pleural invasion of the apex chest wall. **b** Parietal pleurectomy at the site of tumor invasion and additional resection of the extra parietal pleural fatty tissue was performed by complete VATS. *The site of tumor invasion of the chest wall. **Apex of the right upper lobe. ***Superior vena cava

The patient was positioned in the left lateral decubitus position. First, we observed the right thoracic cavity using a thoracoscopy through a 10-mm trocar in the sixth intercostal space on the midaxillary line. Because only partial adhesion of the chest wall apex was observed (Fig. 2a), we judged that tumor resection could be accomplished by complete VATS. We inserted a 7.5-mm trocar in the fourth intercostal space on the posterior axillary line and a 3-cm wound retractor in the same intercostal space on the anterior axillary line. Partial parietal pleurectomy was performed with the resection of the extra parietal pleural fatty tissue of the site of tumor adhesion (Fig. 2b). We confirmed that the extra parietal pleural fatty tissue was a safe margin, ensuring (by an intraoperative diagnosis) that it contained no viable cancer cells. Subsequently, we performed right upper lobectomy with lymph node dissection (ND2a-1) through complete VATS.

**Fig. 3:**
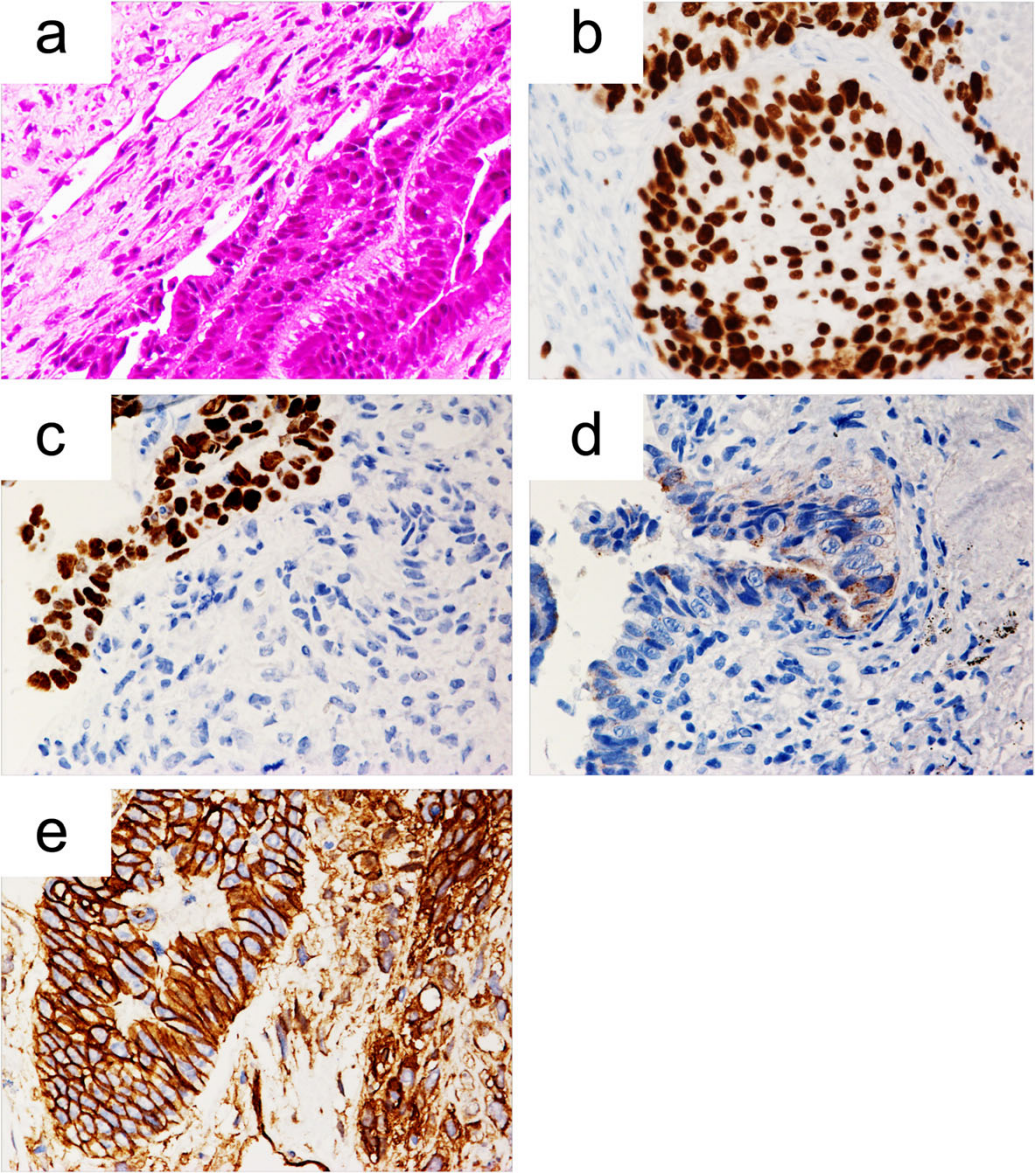
On the histopathological examination, H-FLAC exhibits the absence of morule formation (**a**: H&E staining, × 100 objective lens). Immunohistochemically, the tumor cells were positive for p53 (**b**, × 200 objective lens), SALL4 (**c**, × 200 objective lens), and Glypican3 (**d**, × 200 objective lens). The tumor cells showed membranous staining of β-catenin (**e**, × 200 objective lens)

A postoperative pathological examination revealed no viable cancer cells in the resected tumor. The pathological effect of induction therapy was classified as a pathologically complete cell death, Ef 3. However, extensive fibrotic lesions with hyaline degeneration were confirmed in the resected specimen as the evidence of the effect of chemoradiotherapy. Parietal pleural invasion of the tumor was suggested on histopathological images of the resected specimen in which elastic fibers disappeared and were replaced by connective tissue. We reexamined the preoperative bronchoscopic biopsy specimen, which had been diagnosed as non-small cell lung cancer. A histopathological examination revealed the absence of morule formation (Fig. 3a). Immunohistochemistry revealed that the tumor cells were positive for p53 protein (Fig. 3b), oncofetal protein (SALL4 and Glypican3), and β-catenin (Fig. 3c–e). With respect to β-catenin staining, membranous expression patterns were observed (Fig. 3e). As for neuroendocrine markers, the specimen was positive for CD56 but negative for chromogranin A and synaptophysin. These immunohistochemistry results supported a pathological diagnosis of adenocarcinoma with fetal features (high grade). We decided not to perform adjuvant chemotherapy because of the pathological complete response to neoadjuvant chemoradiotherapy.

The patient was discharged without postoperative complications on the 14th day after the surgery. He receives clinical and radiologic assessments every 3 months. No recurrence was observed at 2 years of follow-up.

## Discussion

We reported a case of adenocarcinoma with fetal features (high grade) invading the superior sulcus that showed a complete response to neoadjuvant chemoradiotherapy, which was followed by right upper lobectomy under complete VATS. This report suggests that neoadjuvant chemoradiotherapy may be effective for H-FLAC. We diagnosed the tumor in this case as adenocarcinoma with fetal features based on new criteria for adenocarcinoma in small biopsies and cytology of the 2015 WHO classification of lung tumors [2]; according to the pathological definition of FLAC, the tumors are composed of columnar atypical cells arranged in a complex glandular pattern, the cytoplasm of which is vacuolated and clear due to the presence of glycogen. The histopathological and immunohistochemical findings in this case were consistent with the features of H-FLAC [1, 3]. Collectively, we recognized the diagnosis of the tumor as H-FLAC. Since H-FLAC often progresses to an advanced stage at presentation, it is known that the prognosis is poor in comparison to L-FLAC. Although there are no standard treatments for FLAC, complete surgical resection is the first choice of management for resectable cases, similarly to all subtypes of primary lung cancer [4]. However, the benefit of chemotherapy and radiotherapy for FLAC is not well defined [5]. In addition, the role of chemotherapy and radiotherapy in the neoadjuvant or adjuvant setting is not well known [4]. Only a few cases of L-FLAC are reported to have been treated with neoadjuvant chemotherapy or adjuvant chemoradiotherapy [6–8]. Although there were few reports of neoadjuvant or adjuvant therapy for H-FLAC, Giusti et al. reported a case in which a multidisciplinary approach was used in the treatment of H-FLAC [9]. They showed a case treated in a neoadjuvant setting with cisplatin plus docetaxel. However, the administration of carboplatin plus vinorelbine and radiotherapy in the adjuvant setting was performed separately because the pathological examination of the resected specimen showed a pathologic partial response. To our knowledge, our case is the first report of H-FLAC showing a pathologic complete response to neoadjuvant concurrent chemoradiotherapy. The reason why the cisplatin plus vinorelbine regimen showed a remarkable effect in our case was unknown. In addition, we could not determine whether this is the most effective regimen because of the small number of reports of neoadjuvant therapy for H-FLAC. Chemotherapy with concurrent radiotherapy may be effective for H-FLAC.

This report also suggests that superior sulcus tumors may be successfully resected by complete VATS lobectomy in cases in which neoadjuvant chemoradiotherapy showed a high degree of efficacy. Preoperative concurrent chemoradiotherapy is recommended as the standard treatment for resectable non-small cell lung cancer of the superior sulcus [10]. However, the optimal regimen and irradiation dose have not been established. Cisplatin plus vinorelbine in preoperative chemotherapy with 40 Gy of radiation was used as neoadjuvant therapy in the present case because it has been reported to be effective for non-small cell lung cancer [11]. It is known that the surgery for superior sulcus tumors generally involves the en bloc resection of the right upper lobe, chest wall, and nerve roots by the posterior approach or an anterior transcervical approach with a long L-shaped incision. Recently, the application of VATS-combined hybrid procedures has been reported in a small number of cases [12, 13]. However, these approaches involve invasive procedures such as rib resection or sternotomy. In the present case, neoadjuvant chemoradiotherapy achieved remarkable tumor reduction. It seems that this enabled the performance of complete VATS lobectomy as a minimally invasive surgical approach. Since the patient in this case had neurological complications, it was clear that the tumor had been affecting the C8 and T1 nerve roots. As the neurological symptoms disappeared due to the remarkable reduction of the tumor after the chemoradiotherapy, it is likely that the tumor had been compressing the nerve roots without direct infiltration of the nerves. Although tumor invasion of the parietal pleura was suggested on histopathological images of the resected specimen in which elastic fibers disappeared and were replaced by connective tissue, it seems that the tumor had not extended beyond the parietal pleura. The outcome of our case suggests that it may be possible to resect a superior sulcus tumor without bony thorax defects by complete VATS.

## Conclusions

We reported an extremely rare case of adenocarcinoma with fetal features (high grade) invading the right superior sulcus, which showed a complete response to neoadjuvant chemoradiotherapy and which was successfully resected by complete VATS. However, the role of neoadjuvant chemoradiotherapy for H-FLAC is not well established. Although the complete resection of superior sulcus tumor without bony thorax defects by VATS alone may be possible after a good response to induction therapy, the optimal procedure remains to be established. This case provides and suggests a useful approach to the management of advanced-stage H-FLAC. More cases are needed to clarify the effects of this treatment method.

## Data Availability

Data sharing is not applicable to this report as no datasets were created or analyzed during the current study.

## Abbreviations

CEA: Carcinoembryonic antigen
CT: Computed tomography
FLAC: Fetal adenocarcinoma
H-FLAC: High-grade fetal adenocarcinoma
L-FLAC: Low-grade fetal adenocarcinoma
MRI: Magnetic resonance imaging
VATS: Video-assisted thoracoscopic surgery
